# Spontaneous Regression and Resolution of Breast Implant-Associated Anaplastic Large Cell Lymphoma: Implications for Research, Diagnosis and Clinical Management

**DOI:** 10.1007/s00266-017-1064-z

**Published:** 2018-02-14

**Authors:** Daniel Fleming, Jason Stone, Patrick Tansley

**Affiliations:** 1 Cosmetic Surgery Institute of Australia, PO Box 213, Fortitude Valley, Brisbane, QLD 4006 Australia; 2 QML Pathology, 1 Riverview Place, Metroplex on Gateway, Murarrie, QLD 4172 Australia; 3 NorthEast Plastic Surgery, Wickham House, Level 1 155 Wickham Terrace, Spring Hill, Brisbane, QLD 4000 Australia

**Keywords:** BIA-ALCL, Epidemiology, Spontaneous, Regression, Resolution

## Abstract

**Background:**

First described in 1997, breast implant-associated anaplastic large cell lymphoma (BIA-ALCL) was recognised by the World Health Organisation in 2016 as a specific disease. It typically presents as a late seroma-containing atypical, monoclonal T cells which are CD30+ and anaplastic lymphoma kinase negative. Until recently, it was thought that the disease was very rare. However, it is being diagnosed increasingly frequently with 56 cases confirmed in Australia by September 2017 and the estimated incidence revised from 1 in 300,000 to between 1 in 1000 and 1 in 10,000 patients with bilateral implants. There is debate about the spectrum of BIA-ALCL. According to the current WHO classification, BIA-ALCL is a cancer in all cases. Treatment guidelines require that it is treated urgently with a minimum of bilateral removal of implants and capsulectomies. Whilst acknowledging the disease has been under diagnosed in the past, with some notable exceptions the BIA-ALCL literature has given scant attention to the epidemiological evidence. Now that it is known that the disease may occur in up to 1 in 1000 patients with a median of 7.5 years from implantation to diagnosis, understanding it in its epidemiological context is imperative. The epidemiology of cancer and lymphoma in women with breast implants strongly suggests that most patients do not have a cancer that will inevitably progress without treatment but instead a self-limiting lympho-proliferative disorder. Although the possibility of spontaneous regression has been raised and the observation made that treatment delay did not seem to increase the risk of spread, the main objection to the lympho-proliferative hypothesis has been the lack of documented cases of spontaneous regression or resolution. Because all cases currently are considered malignant and treated urgently, only case report evidence, interpreted in the proper epidemiological context, is likely to be available to challenge this thinking.

**Methods and Results:**

New observations and interpretation of the epidemiology of BIA-ALCL are made. These are supported by the presentation of two cases, which to the best of our knowledge comprise the first documented evidence of spontaneous regression and spontaneous resolution of confirmed BIA-ALCL.

**Conclusions:**

The epidemiology of the disease strongly suggests that the vast majority of cases are not a cancer that will inevitably progress without treatment. The findings presented in the manuscript provide supportive clinical evidence. Consequently, an alternative view of BIA-ALCL with implications for research, diagnosis and clinical management needs to be considered.

**Level of Evidence IV:**

This journal requires that authors assign a level of evidence to each article. For a full description of these Evidence-Based Medicine ratings, please refer to the Table of Contents or the online Instructions to Authors www.springer.com/00266.

## Introduction

First described in 1997, breast implant-associated anaplastic large cell lymphoma (BIA-ALCL) was recognised by the World Health Organisation in 2016 as a specific disease [1, 2]. It typically presents as a late seroma-containing atypical, monoclonal T cells which are CD30+ and anaplastic lymphoma kinase (ALK)−. Until recently, it was thought that the disease was very rare [3]. However, it is being diagnosed increasingly frequently with 56 cases confirmed in Australia by September 2017 and the estimated incidence revised from 1 in 300,000 to between 1 in 1000 and 1 in 10,000 patients with bilateral implants [4, 5]. There is debate about the spectrum of BIA-ALCL [6, 7]. According to the current WHO classification, BIA-ALCL is a cancer in all cases. Treatment guidelines require it is treated urgently with a minimum of bilateral removal of implants and capsulectomies [8]. Whilst acknowledging the disease has been under diagnosed in the past [8], with some notable exceptions the BIA-ALCL literature has given scant attention to the epidemiological evidence [9, 10]. Now that it is known that the disease may occur in up to 1 in 1000 patients with a median of 7.5 years from implantation to diagnosis, understanding it in its epidemiological context is imperative [5, 11]. The epidemiology of cancer and lymphoma in women with breast implants strongly suggests that most patients do not have a cancer that will inevitably progress without treatment but instead a self-limiting lympho-proliferative disorder [12–17]. Although the possibility of spontaneous regression has been raised and the observation made that treatment delay did not seem to increase the risk of spread [9], the main objection to the lympho-proliferative hypothesis has been the lack of documented cases of spontaneous regression or resolution. Because all cases currently are considered malignant and treated urgently, only case report evidence, interpreted in the proper epidemiological context, is likely to be available to challenge this thinking.

## Materials, Methods and Results

We reviewed the available epidemiology and present two cases, which to the best of our knowledge comprise the first documented evidence of spontaneous regression and spontaneous resolution of confirmed BIA-ALCL.

### Case 1: Spontaneous Regression

In 1994, a 32-year-old female underwent bilateral, subglandular breast augmentation and concomitant periareolar mastopexy using Mentor (Mentor Corp., Santa Barbara, CA), smooth, saline implants. A left sided post-operative wound infection was treated conservatively. She developed bilateral capsular contracture after some months.

In 2009, she underwent bilateral explantation and replacement with Silimed (Silimed, Rio de Janeiro), polyurethane foam-covered silicone gel implants in new submuscular pockets. The left capsule was heavily calcified and excised; the right capsule was retained. Her recovery was uneventful.

**Fig. 1:**
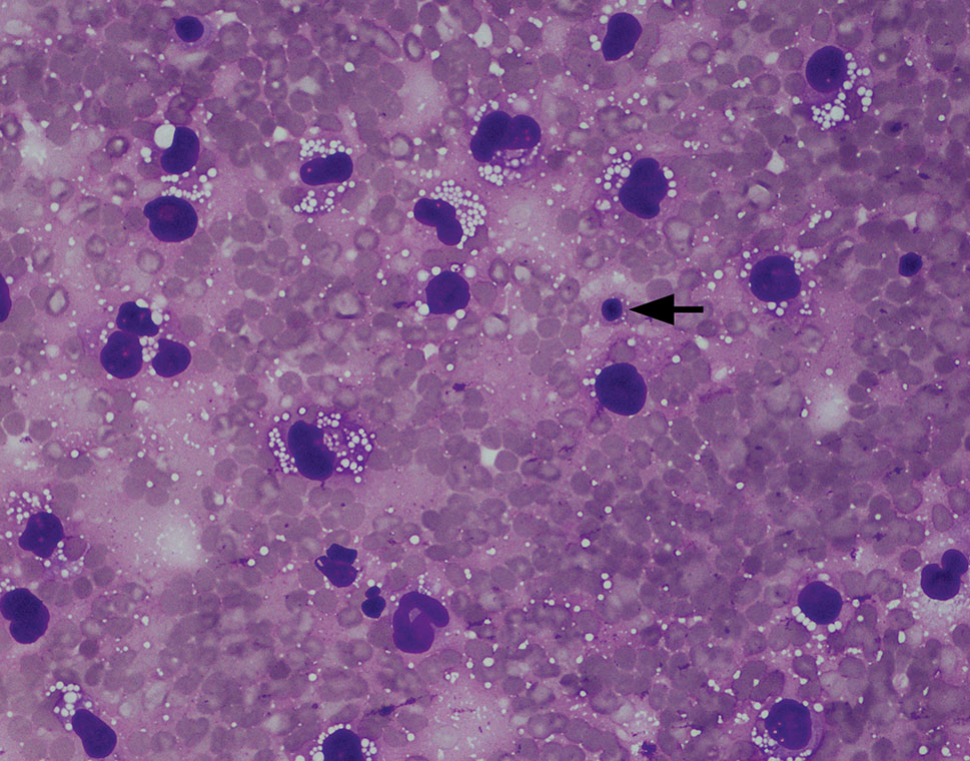
Case 1. Seroma cytology January 2015. Giemsa stain ×400. All the larger cells seen are tumour cells. Atypical cytological features include abundant finely vacuolated cytoplasm, marked nuclear pleomorphism, irregular nuclear membranes and polylobulated nuclei. A normal small lymphocyte (arrowed) allows easy comparison. The background comprises erythrocytes

**Fig. 2:**
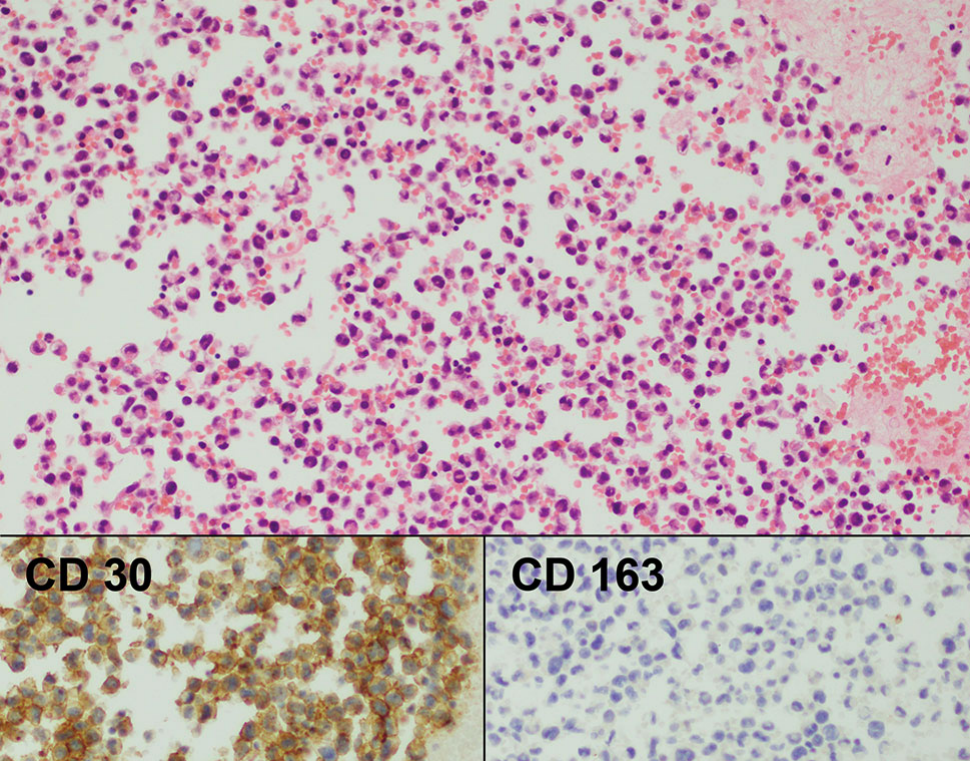
Case 1. Cell block of seroma fluid January 2015. Haematoxylin & Eosin ×100. This lower power magnification demonstrates the marked cellularity of the sample. The inserts show that virtually every cell is positive for CD30 and negative for CD163 (a macrophage marker)

**Fig. 3:**
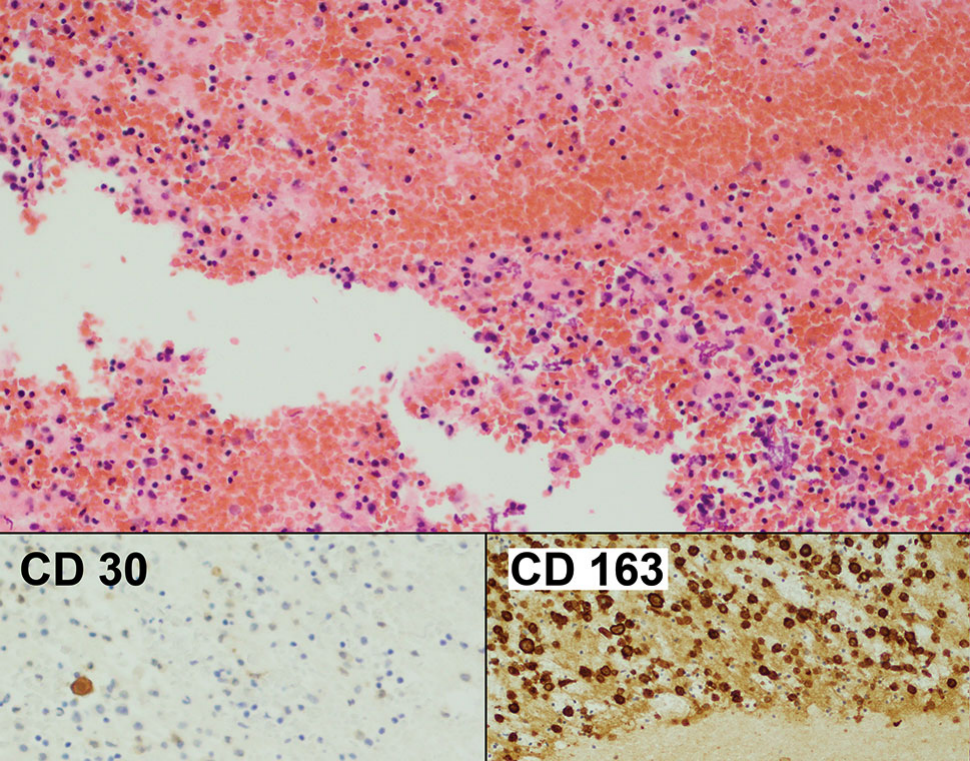
Case 1. Cell block of seroma fluid March 2015. Haematoxylin & Eosin ×100. The inserts show that now there are only scant CD30 positive cells and that most of the cells are CD163 positive benign macrophages

**Fig. 4:**
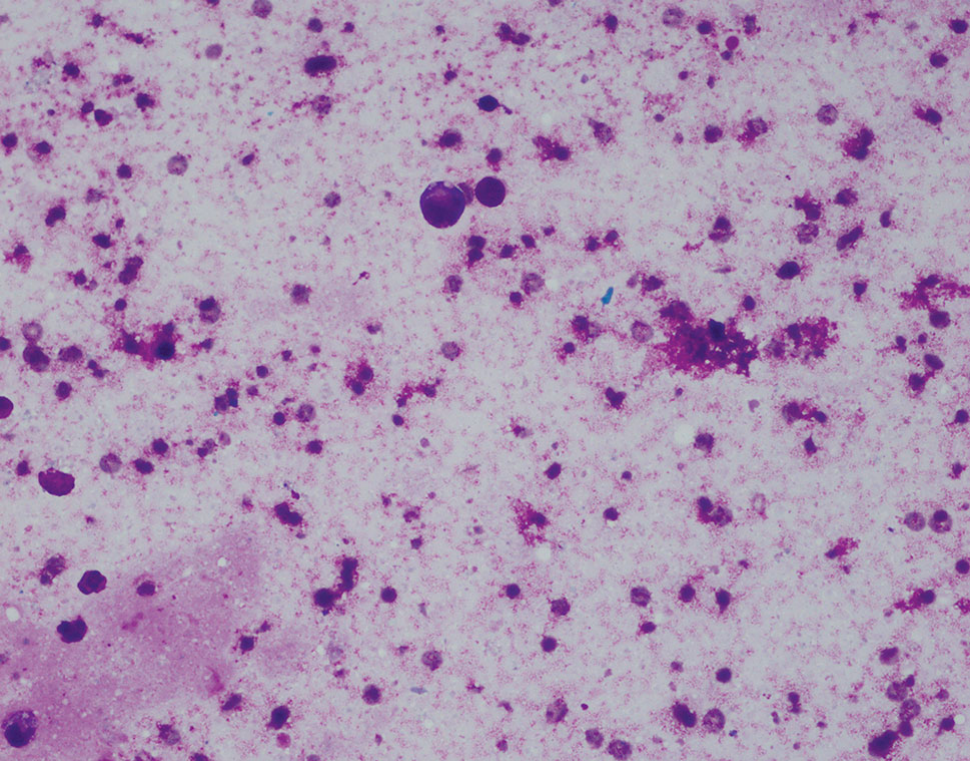
Case 1. Seroma cytology April 2015. Giemsa stain ×400. The fluid is remarkably more paucicellular than previous samples. This photomicrograph shows only scant larger cells with background cellular debris and a few small benign lymphocytes, indicating very low level of residual lymphoma. The sample was too paucicellular for a cell block

**Fig. 5:**
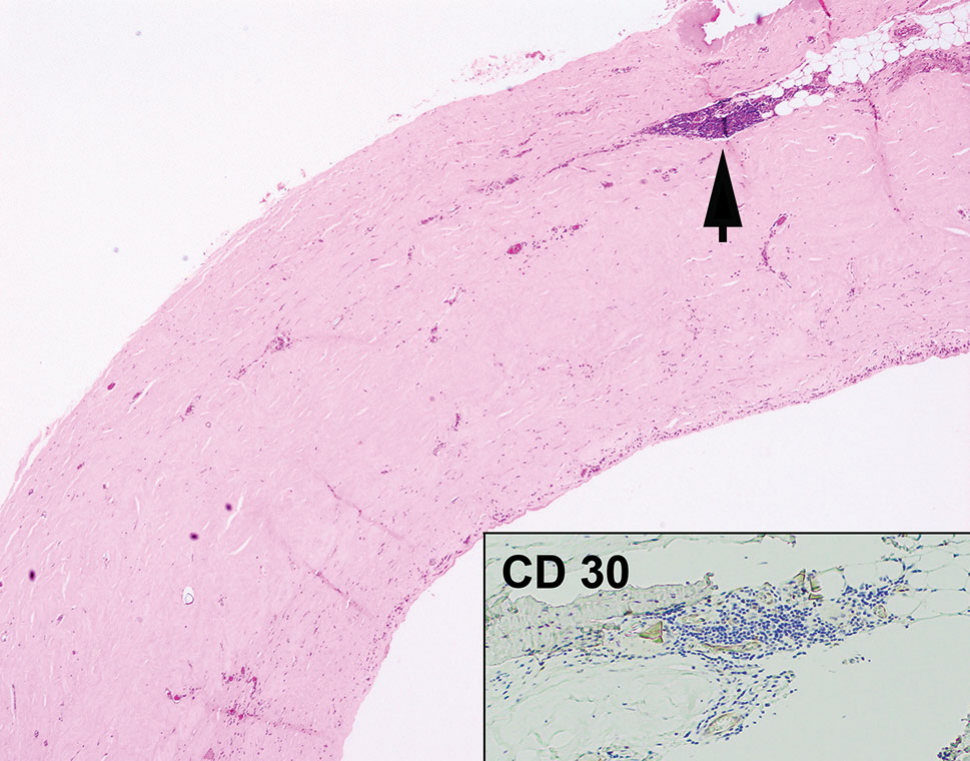
Case 1. Histological section of the implant capsule April 2015. Haematoxylin & Eosin ×100. There is a bland fibrous capsule with occasional reactive lymphoid aggregate (arrowed) and areas of pseudosynovial metaplasia. No capsular lymphoma is present. The inset shows that the reactive lymphoid aggregates are negative for CD30

In May 2014, aged 51, she presented to her GP with a unilateral enlargement of the right breast. An ultrasound scan demonstrated the presence of a large seroma. She delayed seeking treatment for 8 months until January 2015 when she underwent aspiration. Cytology was diagnostic for BIA-ALCL with abundant atypical T cells that were CD30+ and ALK− (Figs. 1, 2). Culture was negative. Subsequent bone marrow aspirate, blood investigations and PET scan were normal. Two months later in March 2015, the aspiration was repeated. Most of the cells identified were benign macrophages and flow cytometry was normal. The very scant CD30+ cells identified (Fig. 3) were interpreted as representing a combination of benign activated lymphocytes, a recognised phenomenon [18–20] and very low level of lymphoma (< 5%). One month later in April 2015, she underwent breast implant explantation and capsulectomy. At surgery, occasional atypical lymphoid cells were identified on cytological examination of the greatly reduced residual seroma fluid (Fig. 4). The fluid was paucicellular, and there was insufficient cellular material for reliable immunohistochemical examination. Flow cytometry was non-diagnostic. Widely sampled histopathology of the capsule was normal, and capsular CD30 was negative (Fig. 5). The patient has since remained asymptomatic.

### Case 2: Spontaneous Resolution

**Fig. 6:**
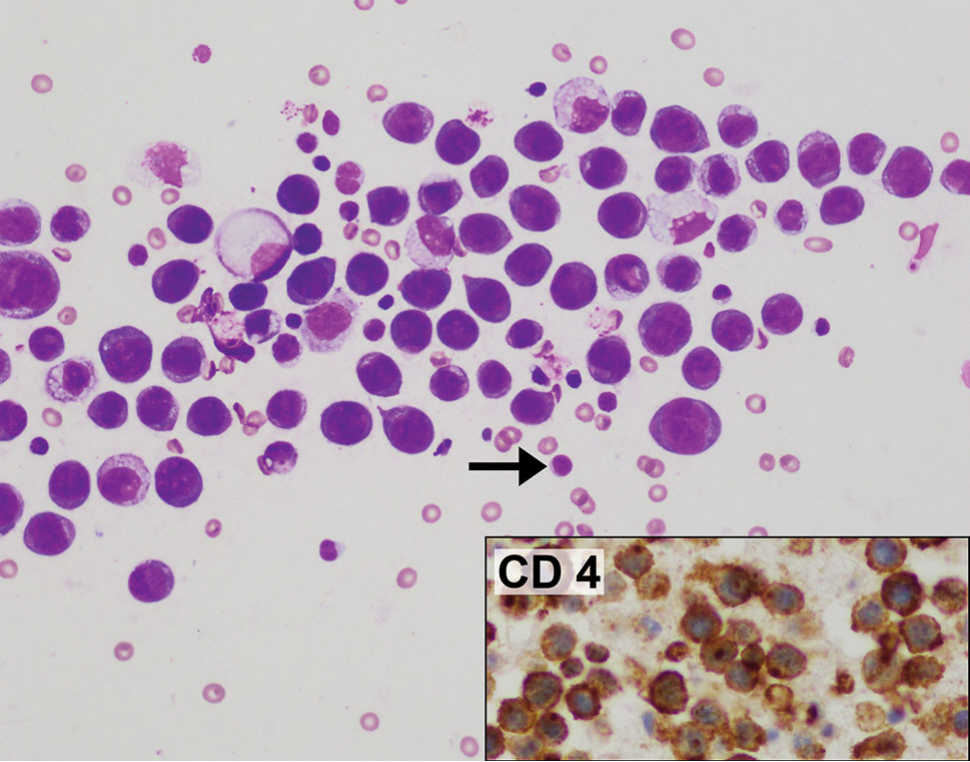
Case 2. Seroma cytology from aspiration on 7 March 2017. Giemsa stain ×400. All the larger cells seen are tumour cells. A normal small lymphocyte (arrowed) allows easy comparison. The insert shows the CD4 positivity

**Fig. 7:**
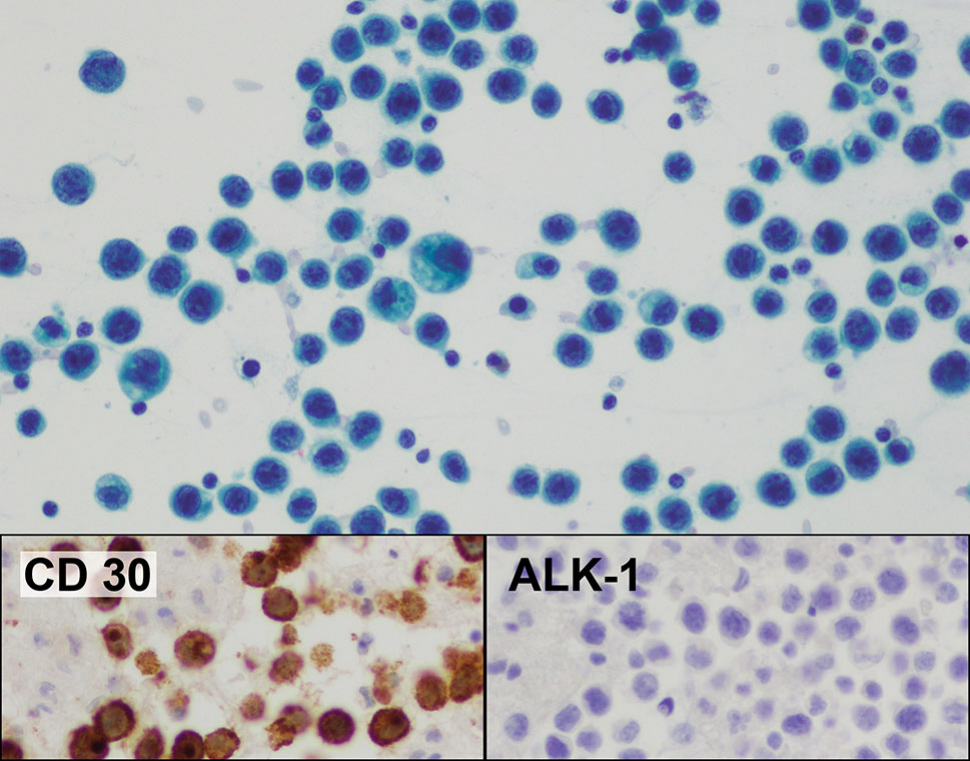
Case 2. Seroma cytology from aspiration on 7 March 2017. Papanicolaou stain ×400. The insert shows the positive CD30 and negative ALK–1 immunocytochemistry

**Fig. 8:**
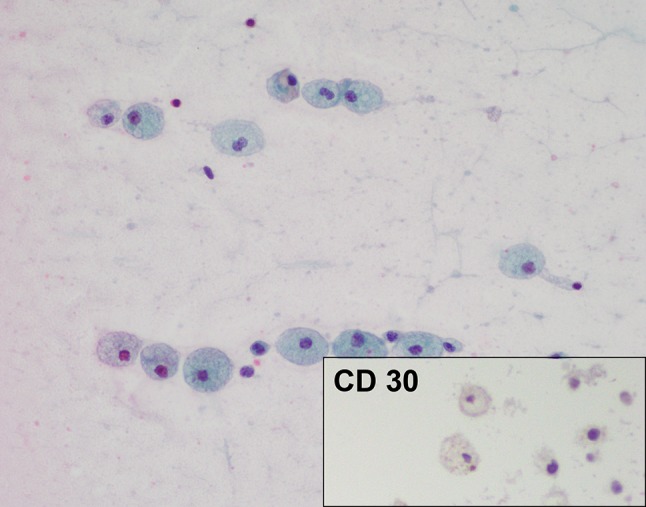
Case 2. Peri-implant fluid at explant on 5 May 2017. Papanicolaou stain ×400. Benign foamy macrophages are present (CD30 negative). No lymphoma cells are seen

**Fig. 9:**
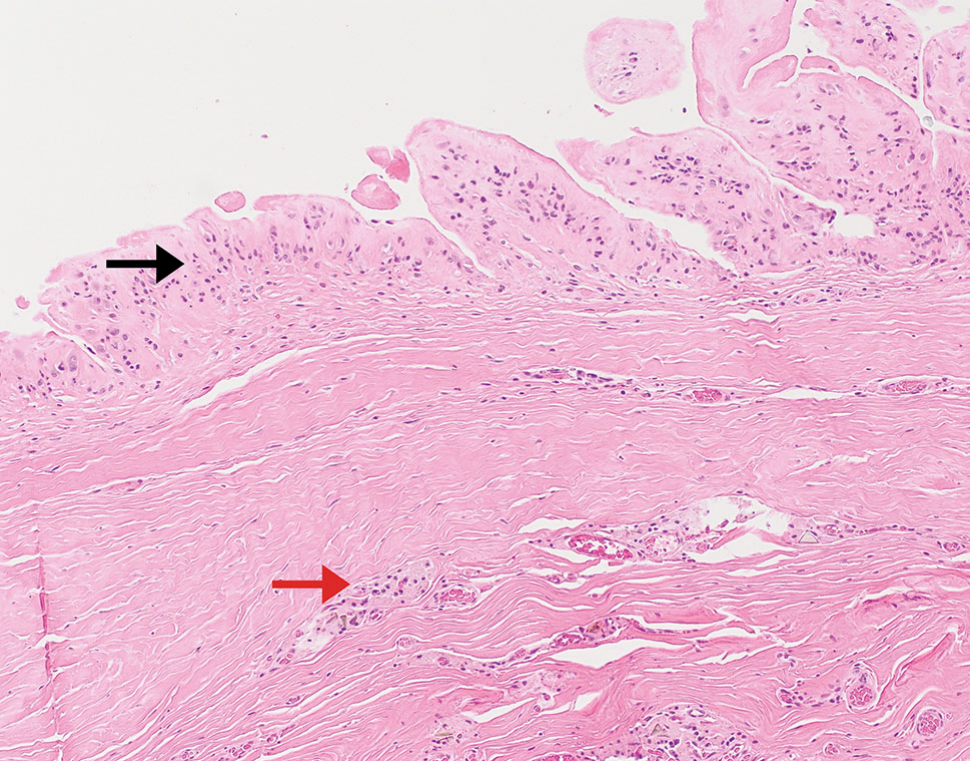
Case 2. Histological section of the removed implant capsule at explantation on 5 May 2017. Haematoxylin & Eosin ×100. There is benign pseudosynovial metaplasia (black arrow) and foreign body reaction to silicone material (red arrow). No lymphoma is seen

In February 2017, a 24-year-old female complained of a sudden enlargement of her left breast. She had had an uncomplicated primary breast augmentation with Silimed polyurethane foam-covered silicone gel filled implants in submuscular pockets in May 2013. An ultrasound reported a seroma with an estimated volume of 50 cc. Aspiration was performed 10 days later. The estimated volume was again 50 cc, but a total of 80 cc was drained. Cytology and immunohistochemistry confirmed BIA-ALCL with atypical T cells which were CD30+ and ALK− (Figs. 6, 7). Culture of the fluid was negative. The patient had no further symptoms. Haematological investigations and a PET scan were normal. The patient was informed that she had a malignancy and underwent bilateral explantation and capsulectomies on 5 May 2017, 10 weeks after the onset of her symptoms. Cytology, flow cytometry and immunohistochemistry of a small residual fluid collection, and histopathology of the capsule, showed no evidence of malignancy (Figs. 8, 9). The patient has remained asymptomatic, and a repeat PET scan on 5 July 2017 was normal.

The slides from both cases were sent for external expert review which confirmed the initial diagnoses and subsequent findings.

## Discussion

The first known case of BIA-ALCL in Australia was diagnosed in 2007, but recently a rapidly rising incidence has been reported [11]. Forty-six cases had been reported by December 2016 in Australia [5], but by September 2017 further 10 cases had been confirmed with 2 more under investigation [4, 21]. The absence of disease in the explanted capsule has not been classified as spontaneous resolution, and seroma-only disease is still classified as a stage 1 malignancy [2]. Relevantly, until recently only capsular tissue and not seroma fluid was submitted for analysis at explantation [22]. Thus, other cases of spontaneous regression or resolution may have been missed. For example, the case of spontaneous resolution presented here would have been missed had the small, asymptomatic residual seroma fluid not been analysed at explantation. The case presented of spontaneous regression is unique because 7 months elapsed from first presentation and confirmation of seroma to the diagnosis being made on aspiration. Also, there was a further 4-month delay before the patient consented to surgical management. Therefore, 11 months elapsed from her presentation with late seroma to surgical explantation. The case has presented us with an opportunity to follow the natural history of BIA-ALCL for almost 1 year. Until and unless treatment guidelines change, it is unlikely to be repeated as patients with confirmed disease are now treated more expediently.

The case of spontaneous resolution occurred within 10 weeks of the patient first having symptoms and 8 weeks after the confirmed diagnosis of BIA-ALCL. This not only proves that the disease can spontaneously resolve but also that it can do so rapidly. The case allows us to reflect on the consequences of the changed management of seroma patients which is based on our relatively new and limited knowledge of BIA-ALCL. Previously, this patient, following a single aspiration and no further symptoms, would not have received any further treatment and would have been diagnosed as a simple seroma. With a symptomatic seroma, estimated as 50 cc on an ultrasound scan, she may or may not have had an aspiration at all. If she did, the fluid would not have been subjected to cytological examination. She would likely have been given antibiotics, and she would not have had any more symptoms just as she did not have any following the diagnosis of BIA-ALCL.

All confirmed BIA-ALCL patients in Australia were exposed to textured or polyurethane covered implants [11]. The median time from implantation to diagnosis was 7.5 years, and 90% of the cases had occurred by 14 years [5, 11]. Textured implants have been widely, and increasingly, used in Australia since 1991 yet the first case in Australia was not recognised until 16 years later. The testing of late seromas for cytology did not commence until 2008 and has become increasingly common since. There is no reason to suppose that BIA-ALCL was not present with the same incidence in textured-implant-related late seromas prior to the advent of cytological testing as afterwards. This begs the question, where are the cases which should have been diagnosed in the interim? Cancer registry data have shown no increase in the incidence of non-Hodgkin lymphoma in women in the period 2000–2013 [23]. The existence of spontaneous regression and spontaneous resolution explains what happened to the seroma patients who had undiagnosed BIA-ALCL prior to the onset of cytological testing to look for it—they got better, often without surgical intervention [24, 25]. The inescapable conclusion is that the rapid and accelerating rise in the diagnosis of BIA-ALCL in Australia is just that—a rise in the diagnosis of the disease, not a rise in its incidence. This would not be unique. Observing the more than sixfold increase in the diagnosis of thyroid cancer without a change in mortality following the onset of screening in South Korea, the authors concluded, “over detection of clinically indolent thyroid cancers is the best explanation for the observed findings in our study” [26].

These findings may not be unexpected as a pathological precedent for spontaneous resolution of a similar disease already exists. The spectrum disorder lymphomatoid papulosis and primary cutaneous ALCL is a rare skin disorder that is considered histologically malignant but often clinically benign [27]. Lesions contain atypical T cells that are also CD30+ and ALK−, as with BIA-ALCL [28]. The disease, which has been recognised since 1968, behaves similarly to BIA-ALCL in that it spreads infrequently and has an excellent prognosis. Importantly, it can spontaneously resolve, even in the primary cutaneous ALCL form [29].

In conjunction with the observed epidemiology of this disease, these findings have implications for how we consider BIA-ALCL.

- At least two types of BIA-ALCL appear to exist—non-invasive seroma-only disease that typically spontaneously resolves or remains indolent and a much rarer invasive malignant disease, usually with a mass present at diagnosis, which does not [7, 14].
- In Australia, the proportion of patients who have seroma-only disease with no evidence of capsular involvement is now 70% and rising as more cases are diagnosed [21].
- Seroma-only BIA-ALCL should be more accurately described as a lympho-proliferative disorder rather than a malignant lymphoma. Our findings add supportive clinical evidence to the calls others have made, on a mortality basis, for an immediate revision of the WHO classification [30].
- Do any seroma-only cases become invasive and can we develop a method to identify truly seroma-only disease without surgery?
- Although falling, currently 30% of cases have some disease in the capsule. In the absence of a mass, is progression inevitable without treatment? These data and the epidemiological evidence suggest otherwise.

Given the potentially fatal consequences of inadequate treatment, and the present inability to be certain that the disease is not invasive without histopathology, bilateral explantation and capsulectomies should remain the current recommended minimum treatment. However, it should be also acknowledged this may be overtreatment for many patients. In this context, we note that of the four deaths attributed to BIA-ALCL in Australia, two were caused by complications of the aggressive treatment that was initially thought to be necessary for all patients [11]. The historical harmful overtreatment of other malignancies, for example prostate cancer and neuroblastoma, has been established and, with increasing knowledge, more conservative strategies safely implemented [31, 32].

The primary goal remains the safe treatment of BIA-ALCL. A method to identify the majority of patients who may be safely managed without surgery, as they were in the past, from the few who cannot, is needed. Further analysis of seroma-only disease from a clinical, cytological and epidemiological perspective should be a priority. It is through a better understanding of the natural history of the spectrum of this disease that we will be able to advise correctly and apply more appropriate therapy for the increasing number of women with this diagnosis, ensuring the vast majority are not told, incorrectly, they have cancer.

## Conclusions

The epidemiology of the disease strongly suggests that the vast majority of cases are not a cancer that will inevitably progress without treatment. The findings presented in the manuscript provide supportive clinical evidence and emphasise the importance of analysing seroma fluid as well as the capsule at explantation. An alternative view of BIA-ALCL with implications for research, diagnosis and clinical management needs to be considered.
